# Research on Waste Plastics Classification Method Based on Multi-Scale Feature Fusion

**DOI:** 10.3390/s22207974

**Published:** 2022-10-19

**Authors:** Zhenxing Cai, Jianhong Yang, Huaiying Fang, Tianchen Ji, Yangyang Hu, Xin Wang

**Affiliations:** Key Laboratory of Process Monitoring and System Optimization for Mechanical and Electrical Equipment (Huaqiao University), Fujian Province University, Xiamen 361021, China

**Keywords:** plastic bottles recycling, hyperspectral image, multi-scale feature fusion

## Abstract

Microplastic particles produced by non-degradable waste plastic bottles have a critical impact on the environment. Reasonable recycling is a premise that protects the environment and improves economic benefits. In this paper, a multi-scale feature fusion method for RGB and hyperspectral images based on Segmenting Objects by Locations (RHFF-SOLOv1) is proposed, which uses multi-sensor fusion technology to improve the accuracy of identifying transparent polyethylene terephthalate (PET) bottles, blue PET bottles, and transparent polypropylene (PP) bottles on a black conveyor belt. A line-scan camera and near-infrared (NIR) hyperspectral camera covering the spectral range from 935.9 nm to 1722.5 nm are used to obtain RGB and hyperspectral images synchronously. Moreover, we propose a hyperspectral feature band selection method that effectively reduces the dimensionality and selects the bands from 1087.6 nm to 1285.1 nm as the features of the hyperspectral image. The results show that the proposed fusion method improves the accuracy of plastic bottle classification compared with the SOLOv1 method, and the overall accuracy is 95.55%. Finally, compared with other space-spectral fusion methods, RHFF-SOLOv1 is superior to most of them and achieves the best (97.5%) accuracy in blue bottle classification.

## 1. Introduction

The common raw materials of disposable plastic bottles are polyethylene terephthalate (PET) and polypropylene (PP). PET and PP plastic bottles are widely used in the beverage-packaging industry due to their advantages of non-friability and safety. More than 480 billion plastic bottles were sold worldwide in 2016 [1]. Not only do waste plastics pollute the environment, but the microplastics produced by them are also more harmful to bio-safety. If waste plastic bottles are not recycled in time, they will take up considerable amounts of land and cause water and soil pollution [2]. Therefore, the recycling of waste plastic bottles is becoming an important issue from the perspective of protecting the environment and improving economic efficiency.

Since plastic bottles of different colors and materials have different recycling values, they are generally classified according to colors [3,4] and materials [5,6,7]. The advanced classification technologies of waste plastic bottles include deep-learning-based computer vision techniques and spectral detection techniques.

According to research conducted in recent years, computer vision techniques based on deep learning are widely used in various engineering fields, such as fruit picking [8] and the fault diagnosis of structures [9]. In the waste-sorting field, classifying plastic bottles based on computer vision is also effective. Jaikumar P et al. used the mask region proposal convolutional neural network (Mask R-CNN) to perform object detection and instance segmentation of waste plastic bottles in a customized dataset containing 192 images. Data augmentation was used to improve the model’s performance in order to address the limitations of small datasets, and the final model achieved a 59.4 mean average precision (mAP) [10]. Some researchers used convolutional neural networks (CNN) and support vector machines (SVM) to classify plastic bottle images in a dataset containing a total of 1100 images and demonstrated that the CNN outperformed the SVM classifier in terms of testing accuracy [11].

There are several types of plastic resins in plastic bottles; if they are mixed during recycling, they will cause serious problems and the product quality will suffer [12]. Therefore, it is critical to classify plastic bottles according to materials. Optical and spectroscopic methods are used to identify the materials of plastics. Masoumi et al. [13] proposed a plastic identification and separation system based on near-infrared light (NIR). This system achieved the separation of polyethylene terephthalate (PET), high-density polyethylene (HDPE), polyvinyl chloride (PVC), polypropylene (PP), and polystyrene (PS) by calculating the reflectance ratio between 1656 nm and 1724 nm. With the widespread use of CNNs, some researchers have started to focus on combining NIR with a CNN to achieve a highly robust and accurate method of plastic classification, while preprocessing methods such as principal component analysis (PCA) are used for the dimensionality reduction of feature vectors [7].

However, the problem faced in waste plastic bottles’ classification is that computer vision technology based on deep learning mainly relies on RGB images. Distinguishing between blue and transparent plastic bottles on a black conveyor belt is difficult due to the influence of the bottles’ quality and the nearby light source. For spectral detection techniques, a disadvantage of NIR is that it is strongly affected by dark colors [14], which not only leads to the shift of reflection but also eliminates the peak value, and hues do not affect spectral reflectance [13,15]. Therefore, although spectral detection techniques can effectively distinguish plastic bottles according to materials, they can not classify them more precisely according to colors.

Light blue PET bottles, transparent PET bottles, and transparent PP bottles on black conveyor belts are difficult to classify via computer vision techniques based on deep learning, and it is difficult to distinguish light blue PET bottles and transparent PET bottles by spectral detection techniques. In this paper, a multi-scale feature fusion method for RGB and hyperspectral images (HSI) based on Segmenting Objects by Locations [16] (RHFF-SOLOv1) is proposed, and the classification accuracy of waste plastic bottles is improved by the feature fusion of RGB and HSI. This paper first introduces the dual camera platform for image acquisition and preprocessing methods. Then, the network structure of RHFF-SOLOv1 is described. In addition, a spectral feature interval selection method is proposed. Finally, the proposed RHFF-SOLOv1 is compared with other spatial-spectral fusion methods. The results show that our method is superior to most of them and achieves the best accuracy of 97.5% in the classification of blue PET bottles.

## 2. Materials and Methods

### 2.1. Samples Preparation and Data Collection

In this study, waste plastic bottles were collected from the recycled products of a company, including blue PET bottles, transparent PET bottles, and transparent PP bottles. Twenty-five samples were collected for each category as training and test datasets. The caps and labels were removed, as these materials are different from the bottle.

**Figure 1:**
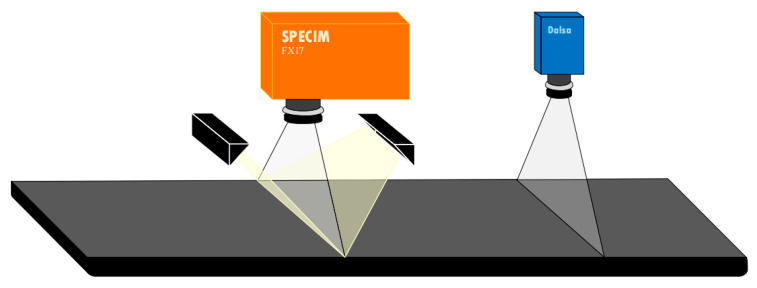
Dual camera platform mode of image acquisition.

For data acquisition, RGB images were collected by a line-scan camera produced by Dalsa, and hyperspectral images were collected by NIR spectral camera with a wavelength range of 935.9–1722.5 nm with 224 bands. In this study, using a variable-speed conveyor as the bottle conveying device, the speed of the belt in the experiment was set to 0.36 m/s. The dual camera platform used for image acquisition is shown in Figure 1.

The dual camera acquisition platform mode of image acquisition was used to collect RGB and hyperspectral images of waste plastic bottles simultaneously. These samples were randomly placed on the conveyor belt and repeatedly collected. In order to address the limitations of custom datasets, we also tried to improve the generalization performance of our model by using data augmentation and Copy-Paste [17]. The final datasets included 768 images in the training set and 300 images in the test set. The size of RGB images is 640 × 640, while the size of hyperspectral images is 640 × 640 pixels with 224 bands in the wavelength range of 935.9–1722.5 nm. According to the color and material of plastic bottles, they were divided into three categories: transparent PET bottles (Trans_PET), blue PET bottles (Blue_PET), and transparent PP bottles (Trans_PP). All experiments were conducted on a workstation equipped with a Giga Texel Shader eXtreme (GTX) 3090 graphics-processing unit (GPU) and 24 G of memory.

### 2.2. Data Processing

The spectrum data of samples is easily affected by various factors, such as the light source and the background. In order to eliminate the effects of dark current and the noise caused by the uneven intensity of the light source, it is necessary to use the black-and-white correction method to convert the collected data into reflectivity, as shown in Formula (1):

(1) $$r=\frac{DN-DN_{b}}{DN_{w}-DN_{b}}$$

where *DN* denotes the collected raw data, while *DN_w_* denotes the standard white frame data, which is obtained by collecting standard whiteboards. *DN_b_* represents the standard black frame data obtained by covering the camera lens, and *r* denotes the obtained reflectivity data.

### 2.3. Network Structure of RHFF-SOLOv1

**Figure 2:**
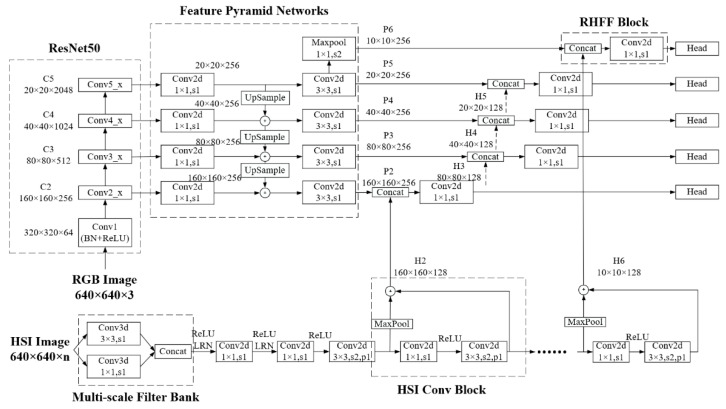
Network structure of RHFF-SOLOv1.

The proposed multi-scale feature fusion method for RGB and hyperspectral images based on SOLOv1 (RHFF-SOLOv1) consists of feature extraction and fusion. In terms of feature extraction, Resnet50 and feature pyramid networks (FPN) are used as the backbone network for the RGB imagery feature extraction branch, and in the branch of hyperspectral image feature extraction, “Multi-scale Filter Bank” [18] is used to obtain the spatial-spectral feature map, which is input into the hyperspectral imagery convolutional neural network (HSI-CNN) to extract multi-scale feature maps. In terms of feature fusion, the feature maps output by FPN and HSI-CNN is concatenated according to its size. Finally, the fused feature maps are input into the detection head of SOLOv1 to predict the position and category of plastic bottles. The proposed RHFF-SOLOv1 integrates multi-scale features of RGB and HSI, and the classification accuracy of plastic bottles is improved. The network structure of RHFF-SOLOv1 is shown in Figure 2.

#### 2.3.1. RGB Imagery Feature Extraction Branch

Resnet50 and FPN are used as the backbone network of the RGB image feature extraction branch, while the output from the second feature extraction stage to the fifth feature extraction stage of Resnet50 is connected with FPN, and FPN outputs multi-scale feature maps. This has the advantage of combining low-detail level information with high-level semantic information.

#### 2.3.2. Hyperspectral Imagery Feature Extraction Branch

**Figure 3:**
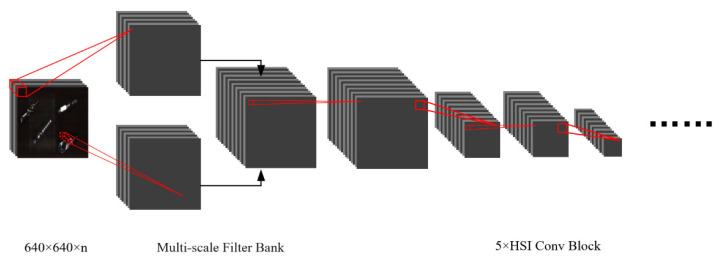
Network structure of HSI-CNN.

In the hyperspectral imagery feature extraction branch, a 3D CNN block with a kernel of n × 3 × 3 and n × 1 × 1 is used to form a “Multi-scale Filter Bank”, where the former is used to extract spatial information, and the latter is used to extract spectral information. Then, the spatial-spectral feature map is used as the input of HSI-CNN, which contains five hyperspectral image convolution blocks (HSI Conv Block). The network structure of HSI CNN is shown in Figure 3.

#### 2.3.3. Multi-Scale Feature Fusion and Detection Head

**Figure 4:**
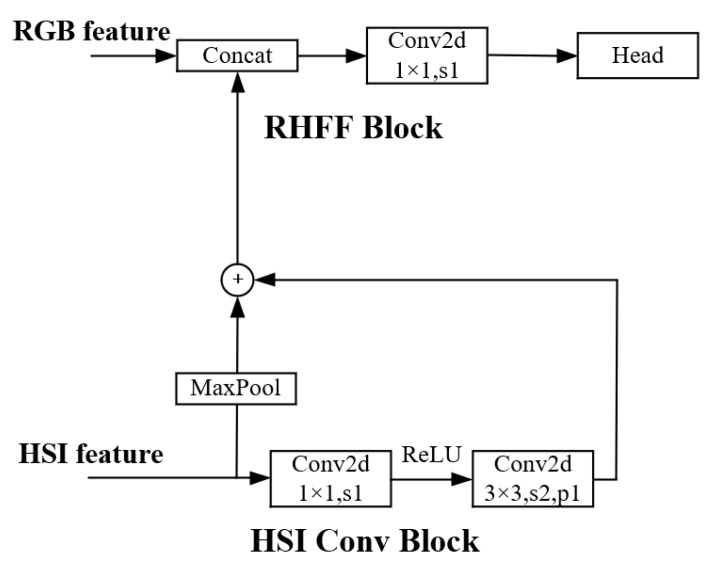
HSI Conv Block and RHFF Block.

Before feature fusion, maximum pooling is used to compress the shallow feature map that is output by HSI Conv Block, and it is added to the deep feature map. The feature fusion method of RGB and hyperspectral images is used to concatenate the feature maps output from FPN and HSI-CNN by size. The multi-scale feature fusion maps are used as the input of the detection head of SOLOv1 and a 2D CNN layer with the kernel of 1 × 1 is used to reduce the channel from 384 to 256. The HSI Conv Block and RHFF Block are shown in Figure 4.

**Figure 5:**
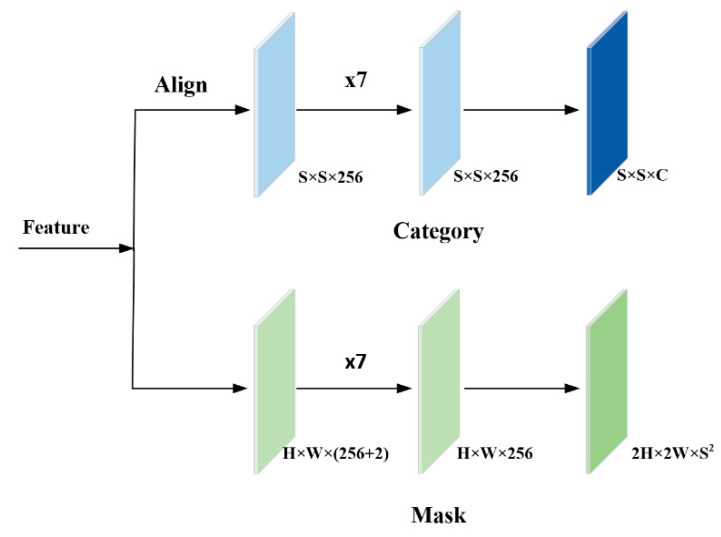
Detection head of SOLOv1.

The detection head of SOLOv1 divides the image into S × S grids; the grid where the center of the instance is located is responsible for predicting the instance category and mask, so the output of the detection head has a category branch and mask branch. The category branch predicts the category of the instance, and the final output is S × S × C, where C represents the number of classes. In the mask branch, the mask of the instance is predicted by the decoupled head, which predicts X and Y to obtain the mask through an “Element-Wise” operation. The detection head of SOLOv1 is shown in Figure 5.

## 3. Results

### 3.1. Hyperspectral Band Selection

In a hyperspectral image, each pixel includes 224 bands and contains many spectral features that will increase the complexity of the model. Therefore, reducing the number of spectral bands in hyperspectral images is necessary.

**Figure 6:**
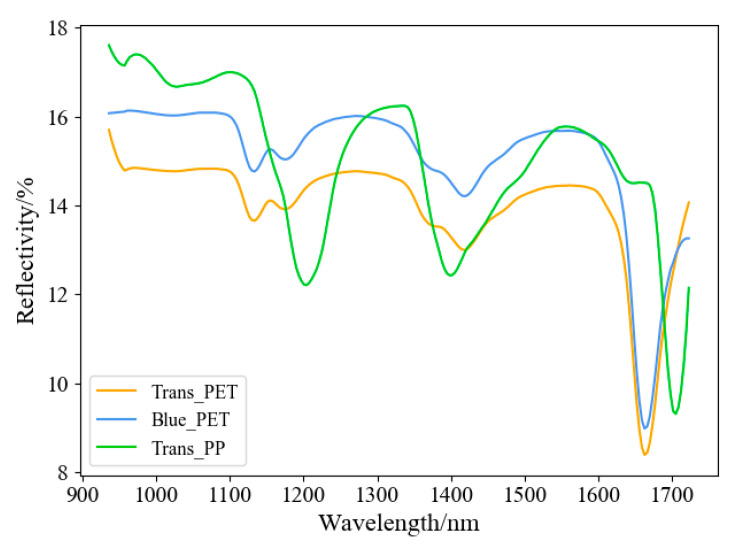
Mean spectral curve of waste plastic bottles.

Figure 6 shows the average spectral curves of Tans_PET, Blue_PET, and Trans_PP. It can be seen that the spectral curves of Trans_PET and Blue_PET are similar, and only the peaks are different. Moreover, the spectral curve of Trans_PP exhibits a large fluctuation amplitude, and it has two obvious absorption valleys at 1200.5 nm and 1394.5 nm. In this paper, a method of hyperspectral feature band selection is proposed, 224 bands are divided into several spectral intervals, and the interval with the most apparent feature is selected.

**Figure 7:**
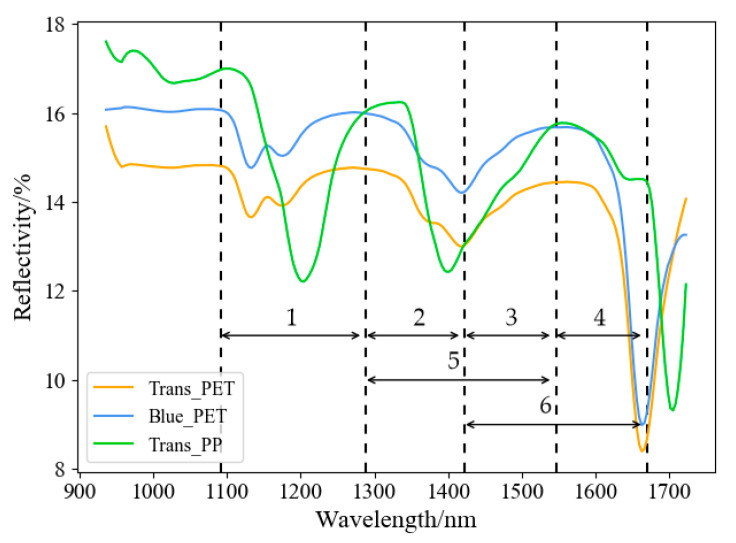
Spectral feature intervals. Interval 1: 1087.6 nm–1285.1 nm; Interval 2: 1285.1 nm–1419.2 nm; Interval 3: 1419.2 nm–1542.6 nm; Interval 4: 1542.6 nm–1666.1 nm; Interval 5: 1285.1 nm–1542.6 nm; Interval 6: 1419.2 nm–1666.1 nm.

Specifically, the mean spectral curves of the waste plastic bottles were smoothed by the Savitzky-Golay filter, and the extreme points were calculated. In order to find the spectral band with the best classification result among the three types, several split points were chosen from among the extreme points, including 1087.6 nm, 1285.1 nm, 1419.2 nm, 1542.6 nm, and 1666.1 nm. The spectral feature intervals are shown in Figure 7.

The bands before 1087.6 nm and after 1666.1 nm were not retained due to noise and inconspicuous features. Finally, different classifiers such as SVM, 1D convolutional neural networks (1D-CNN), and Random Forest (RF) were used to classify the plastic bottles based on the spectral feature intervals. Table 1 shows the overall accuracy of the classification results (%).

The results show that the classification performance is better when interval 1 (1087.6 nm–1285.1 nm) and interval 6 (1419.2 nm–1666.1 nm) are selected, which means that these feature intervals contain important features for the classification of Trans_PET, Blue_PET, and Trans_PP.

### 3.2. Evaluating Indicators

The indicators used to evaluate the classification results include precision (*P*), recall (*R*), overall accuracy (*OA*), average accuracy (*AA*), and Kappa (*k*). The calculation methods are shown in Formulas (2)–(7). TP is the True Positive, FN is the False Negative, TN is the True Negative, and FP is the False Positive.

(2) $$P=\frac{\mathrm{TP}}{\mathrm{TP}+\mathrm{FP}}\times 100\%$$

(3) $$R=\frac{\mathrm{TP}}{\mathrm{TP}+\mathrm{FN}}\times 100\%$$

(4) $$OA=\frac{\mathrm{TP}}{n}\times 100\%$$

(5) $$AA=\frac{\mathrm{sum}(R)}{C}\times 100\%$$

(6) $$k=\frac{p_{o}-p_{e}}{1-p_{e}}\times 100\%$$

(7) $$P_{e}=\frac{\overset{C}{\underset{i=1}{\sum }}a_{i}\times b_{i}}{n^{2}}$$

in which *C* is the number of classes, *n* is the total number of samples, the number of samples of each category is *a_i_*, and the number of prediction samples of each category is *b_i_*. In Formula (6), *p_o_* is *OA*, and *k* is used to evaluate the consistency between the predicted results and the actual classification results.

### 3.3. Comparison between Different Intervals

In order to select the best spectral feature interval, this study used the hyperspectral image of feature intervals 1 and 6 as the input of RHFF-SOLOv1 for comparative experiments. The HSI size of interval 1 is 640 × 640 × 57, and the HSI size of interval 6 is 640 × 640 × 71. The comparison of the experimental results is shown in Table 2.

The results show that the characteristic of spectral interval 1 is better than that of spectral interval 6; so, interval 1 was selected as the hyperspectral image for this study.

### 3.4. Comparison between RHFF-SOLOv1 and Other Methods

In order to verify the feasibility and effectiveness of RHFF-SOLOv1, this paper first compares the classification results of SOLOv1 and RHFF-SOLOv1. Table 3 shows the comparative experimental results (%).

Compared with SOLOv1, the results of OA and AA of the proposed RHFF-SOLOv1 were improved by 8.89% and 8.18%, respectively.

Recently, a large number of studies have applied CNNs to hyperspectral image classification, and these methods can be applied to different applications. Our proposed method is compared with the spatial-spectral fusion networks such as ContextualNet [18], 3D-CNN [19], the Spectral-Spatial Residual Network (SSRN) [20], and Hybrid SpectralNet (HybridSN) [21] to demonstrate that RHFF-SOLOv1 is also superior to spectral detection technology. Since these networks are based on the classification of pixels in HSI, in order to ensure the same number of prediction instances in all experiments, 300 hyperspectral images based on interval 1 in the test set are selected as their datasets, with a size of 640 × 640 × 57. Each HSI is divided into pixels with a ratio of Dataset_train_:Dataset_test_ = 3:7, and the class results with the most pixels in the instance constitute the category of the instance. The comparison of the experimental results (%) is shown in Table 4.

From the results, the proposed RHFF-SOLOv1 improves the OA, AA, and Kappa of waste plastic bottles’ classification results by fusing the hyperspectral image feature map and RGB feature map. Compared with other spatial-spectral fusion networks, RHFF-SOLOv1 is superior, except with respect to SSRN in the evaluation indicators. Among them, RHFF-SOLOv1 achieves the highest accuracy in terms of accuracy for determining blue PET bottles.

**Figure 8:**
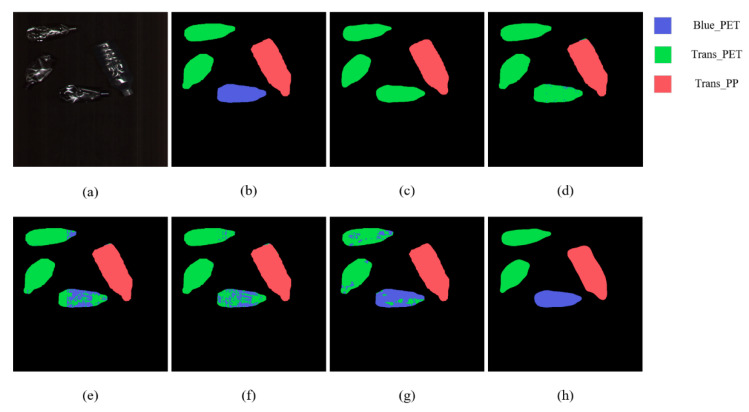
Classification maps for bottles. (**a**) RGB image. (**b**) Ground truth. (**c**–**h**) Predicted classification maps for SOLOv1, ContextualNet, 3D-CNN, SSRN, HybridSN, and proposed RHFF-SOLOv1, respectively.

Figure 8 shows the classification maps for the bottles using SOLOv1, ContextualNet, 3D-CNN, SSRN, HybridSN, and RHFF-SOLOv1. It can be seen that the prediction charts obtained by HybridSN and RHFF-SOLOv1 are of good quality and can correctly identify blue PET bottles more efficiently than other methods.

**Figure 9:**
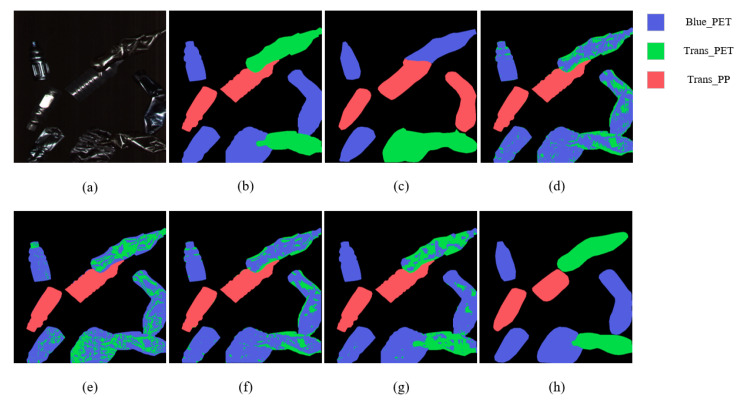
Classification maps for cluttered bottles. (**a**) RGB image. (**b**) Ground truth. (**c**–**h**) Predicted classification maps for SOLOv1, ContextualNet, 3D-CNN, SSRN, HybridSN, and proposed RHFF-SOLOv1, respectively.

The proposed RHFF-SOLOv1 can also achieve good classification results when waste plastic bottles are cluttered and stacked. However, compared to other methods, our method cannot segment the contours of some plastic bottles completely during the segmentation of the instances. The classification maps of the cluttered bottles are shown in Figure 9.

Alongside improving the classification accuracy by multi-scale feature fusion, the determination of a method for more accurately segmenting the contours of the instances is also a future research direction.

## 4. Conclusions

This study proposes a multi-scale feature fusion method of RGB and hyperspectral images based on SOLOv1 (RHFF-SOLOv1). The spectral interval 1 (1087.6 nm–1285.1 nm) was selected as the spectral feature of waste plastic bottles classification through the hyperspectral band selection method. To effectively combine spatial and spectral information, multi-scale feature maps of RGB and HSI were extracted and integrated using RHFF-SOLOv1. In terms of bottle classification accuracy, our proposed method achieves the best (97.5%) accuracy with respect to blue PET bottle classification only. However, the OA, AA, and Kappa results of the proposed method are better than SOLOv1 and the state-of-the-art spatial-spectral fusion network, except with respect to SSRN, which confirms the feasibility and effectiveness of RHFF-SOLOv1. With the development of multi-sensor fusion technology, multiple sources of information can be integrated by effective fusion methods to address the difficulty of classification.

## Figures and Tables

**Table 1:** The overall accuracy of classification results based on feature intervals.

Classifiers	Interval 1	Interval 2	Interval 3	Interval 4	Interval 5	Interval 6
SVM	71.9	70.0	59.5	70.8	70.6	75.7
1D-CNN	88.4	81.8	74.7	79.2	84.8	86.4
RF	76.3	75.6	68.5	73.6	75.4	77.3

**Table 2:** RHFF-SOLOv1 classification results (%) based on different spectral feature intervals.

	Interval 1	Interval 6
Blue_PET	97.60	97.60
Trans_PET	94.80	94.12
Trans_PP	96.00	95.43
OA	95.55	95.01
AA	95.69	95.06
Kappa × 100	93.28	92.47

**Table 3:** The classification results of SOLOv1 and RHFF-SOLOv1.

	SOLOv1	RHFF-SOLOv1
Blue_PET	87.80	97.60
Trans_PET	87.88	94.80
Trans_PP	92.16	96.00
OA	86.66	95.55
AA	87.51	95.69
Kappa × 100	80.10	93.28

**Table 4:** Classification results between RHFF-SOLOv1 and other fusion methods.

	ContextualNet	3D-CNN	SSRN	HybridSN	RHFF-SOLOv1
Blue_PET	79.02	89.89	93.26	92.11	97.60
Trans_PET	86.43	94.49	95.67	98.11	94.80
Trans_PP	97.47	99.48	98.99	96.48	96.00
OA	86.52	94.07	95.69	95.42	95.55
AA	87.68	94.44	96.06	95.69	95.69
Kappa × 100	75.59	91.01	93.47	93.06	93.28

## Data Availability

The data presented in this study are available on request from the corresponding author.
